# Supplementary material for: Familiarity facilitates feature-based face processing
Source: PLoS One. 2017 Jun 5;12(6):e0178895. doi: 10.1371/journal.pone.0178895 (PMC5459439; doi:10.1371/journal.pone.0178895)
Supplement: S1 File — (PDF) [file pone.0178895.s001.pdf]

Supplementary Material

Familiarity Facilitates Feature-based Face Processing

Matteo Visconti di Oleggio Castello*, Kelsey G. Wheeler, Carlo Cipolli, M. Ida Gobbini*

*** Correspondence:**

M. Ida Gobbini

mariaida.gobbini@unibo.it; maria.i.gobbini@dartmouth.edu

Matteo Visconti di Oleggio Castello

mvdoc.gr@dartmouth.edu

Supplementary Table 1. Analysis of Deviance (Wald's χ^2 test) on the Logit Mixed-Model on Accuracies for Target Present trials. Bold and italic typeface show significant terms.

ACCURACY - TARGET PRESENT				
	**Term**	**χ^2**	**df**	**P**
**Main Effects**				
	Set Size	75.01	2	< .001
	Familiarity	0.22	1	0.64
	Target Orientation	19.37	1	< .001
	Target Sex	18.73	1	< .001
**Two-way interactions**				
	Set Size x Familiarity	0.23	2	0.89
	Set Size x Target Orientation	4.78	2	09
	Familiarity x Target Orientation	0.42	1	0.52
**Three-way interaction**				
	Set Size x Familiarity x Target Orientation	3.24	2	0.20

Supplementary Table 2. Analysis of Deviance (Wald's χ^2 test) on the Logit Mixed-Model on Accuracies for Target Absent trials. Bold and italic typeface show significant terms.

ACCURACY - TARGET ABSENT				
	**Term**	**χ^2**	**df**	**P**
**Main Effects**				
	**Set Size**	25.54	2	< .001
	Familiarity	6.75	1	< .01
	Target Orientation	16.54	1	< .001
	Target Sex	3.57	1	0.06
**Two-way interactions**				
	Set Size x Familiarity	0.04	2	0.98
	Set Size x Target Orientation	1.48	2	0.48
	Familiarity x Target Orientation	0.00	1	0.95
**Three-way interaction**				
	Set Size x Familiarity x Target Orientation	0.72	2	0.70

Supplementary Table 3. Analysis of Deviance (Wald's χ^2 test) on the Linear Mixed-Model on Reaction Times for Target Present trials. Bold and italic typeface show significant terms.

RT - TARGET PRESENT				
	**Term**	**χ^2**	**df**	**P**
**Main Effects**				
	Set Size	1318.93	2	< .001
	Familiarity	169.61	1	< .001
	Target Orientation	400.49	1	< .001
	Target Sex	7.96	1	< .01
**Two-way interactions**				
	Set Size x Familiarity	8.59	2	< .05
	Set Size x Target Orientation	2.75	2	0.25
	Familiarity x Target Orientation	9.16	1	< .01
**Three-way interaction**				
	Set Size x Familiarity x Target Orientation	11.17	2	< .01

Supplementary Table 4. Analysis of Deviance (Wald's χ^2 test) on the Linear Mixed-Model on Reaction Times for Target Absent trials. Bold and italic typeface show significant terms.

RT - TARGET ABSENT				
	**Term**	χ^2	**df**	**P**
**Main Effects**				
	Set Size	8131.39	2	< .001
	Familiarity	414.31	1	< .001
	Target Orientation	792.64	1	< .001
	Target Sex	4.99	1	< .05
**Two-way interactions**				
	Set Size x Familiarity	6.59	2	< .05
	Set Size x Target Orientation	6.20	2	< .05
	Familiarity x Target Orientation	6.75	1	< .01
**Three-way interaction**				
	Set Size x Familiarity x Target Orientation	0.56	2	0.75
